# Continuous passive paracentesis versus large-volume paracentesis in the prevention and treatment of intra-abdominal hypertension in the critically ill cirrhotic patient with ascites (COPPTRIAHL): study protocol for a randomized controlled trial

**DOI:** 10.1186/s13063-023-07541-4

**Published:** 2023-08-15

**Authors:** Rui Antunes Pereira, Daniel Virella, Rui Perdigoto, Paulo Marcelino, Faouzi Saliba, Nuno Germano

**Affiliations:** 1 Unidade de Cuidados Intensivos Polivalente 7, Hospital de Curry Cabral, Centro Hospitalar Universitário Lisboa Central, Lisboa, Portugal; 2 Unidade Funcional de Neonatologia, Centro Hospitalar Universitário Lisboa Central, Lisboa, Portugal; 3 Unidade de Transplante, Hospital de Curry Cabral, Centro Hospitalar Universitário Lisboa Central; Nova Medical School, Lisboa, Portugal; 4 Unidade de Cuidados Intensivos Polivalente 4, Hospital de Santa Marta, Centro Hospitalar Universitário Lisboa Central, Lisboa, Portugal; 5 Hôpital Paul Brousse, Hepato-Biliary Center, Université Paris Saclay, INSERM Unit 1193, Villejuif, France

**Keywords:** Abdominal perfusion pressure, Abdominal compartment syndrome, Outcome, Acute kidney injury, Renal failure, Multi-organ failure, Randomized controlled trial

## Abstract

**Background:**

Critically ill patients with cirrhosis and ascites are at high risk for intra-abdominal hypertension (IAH) which increases mortality. Clinical guidelines recommend maintaining intra-abdominal pressure (IAP) below 16 mmHg; nonetheless, more than three quarters of critically ill patients with cirrhosis develop IAH during their first week of ICU stay. Standard-of-care intermittent large-volume paracentesis (LVP) relieves abdominal wall tension, reduces IAP, optimizes abdominal perfusion pressure, and is associated with short-term improvement in renal and pulmonary dysfunction. However, there is no evidence of the superiority of different paracentesis strategies in the prevention and treatment of IAH in critically ill patients with cirrhosis.

This trial aims to compare the outcomes of continuous passive paracentesis versus LVP in the prevention and treatment of IAH in patients with cirrhosis and ascites.

**Methods:**

An investigator-initiated, open label, randomized controlled trial, set in a general ICU specialized in liver disease, was initiated in August 2022, with an expected duration of 36 months. Seventy patients with cirrhosis and ascites will be randomly assigned, in a 1:1 ratio, to receive one of two methods of therapeutic paracentesis. A stratified randomization method, with maximum creatinine and IAP values as strata, will homogenize patient baseline characteristics before trial group allocation, within 24 h of admission.

In the control group, LVP will be performed intermittently according to clinical practice, with a maximum duration of 8 h, while, in the intervention group, continuous passive paracentesis will drain ascitic fluid for up to 7 days.

The primary endpoint is serum creatinine concentration, and secondary endpoints include IAP, measured creatinine clearance, daily urine output, stage 3 acute kidney injury and multiorgan dysfunction assessed at day 7 after enrollment, as well as 28-day mortality rate and renal replacement therapy-free days, and length-of-stay. Prespecified values will be used in case of renal replacement therapy or, beforehand ICU discharge, liver transplant and death. Safety analysis will include paracentesis-related complication rate and harm. Data will be analyzed with an intention-to-treat approach.

**Discussion:**

This is the first trial to compare the impact of different therapeutic paracentesis strategies on organ dysfunction and outcomes in the prevention and treatment of IAH in critically ill patients with cirrhosis and ascites.

**Trial registration:**

ClinicalTrials.gov NCT04322201. Registered on 20 December 2019

**Supplementary Information:**

The online version contains supplementary material available at 10.1186/s13063-023-07541-4.

## Administrative information

**Table:** 

Title {1}	Continuous passive paracentesis versus large-volume paracentesis in the prevention and treatment of intra-abdominal hypertension in the critically ill cirrhotic patient with ascites. (COPPTRIAHL)
Trial registration {2a and 2b}	ClinicalTrials.gov Identifier: NCT04322201 All items from the WHO trial registry are found within the protocol
Protocol version {3}	Protocol version 10.4, January 2022
Funding {4}	This trial has been supported by the *Centro de Investigação do Centro Hospitalar Universitário Lisboa Central* with the trial grant “Fundo de Financiamento de Investigação – FFI06/2019”
Author details {5a}	^1^ Rui Antunes Pereira. Unidade de Cuidados Intensivos Polivalente 7, Hospital de Curry Cabral, Centro Hospitalar Universitário Lisboa Central, Lisboa, Portugal. Corresponding author: email address < rui.m.pereira@chlc.min-saude.pt > , telephone contact + 351 934,341,322 ^2^ Daniel Virella. Unidade Funcional de Neonatologia, Centro Hospitalar Universitário Lisboa Central, Lisboa, Portugal. < danielvirella@chlc.min-saude.pt > ^3^ Rui Perdigoto. Unidade de Transplante, Hospital de Curry Cabral, Centro Hospitalar Universitário Lisboa Central; Nova Medical School, Lisboa, Portugal. < rui.perdigoto@gmail.com > ^4^ Paulo Marcelino. Unidade de Cuidados Intensivos Polivalente 4, Hospital de Santa Marta, Centro Hospitalar Universitário Lisboa Central, Lisboa, Portugal. < p.marcelino@netcabo.pt > ^5^ Faouzi Saliba. Hôpital Paul Brousse, Hepato-Biliary center; Université Paris Saclay; INSERM unit 1193, Villejuif, France. < faouzi.saliba@aphp.fr > ^6^ Nuno Germano. Unidade de Cuidados Intensivos Polivalente 7, Hospital de Curry Cabral, Centro Hospitalar Universitário Lisboa Central, Lisboa, Portugal. < nuno.m.germano@gmail.com >
Name and contact information for the trial sponsor {5b}	Centro Hospitalar Universitário Lisboa Central, E.P.E. Rua da Beneficência, n° 8, 1069–166 Lisboa. Telephone: + 351 217,924,200
Role of sponsor {5c}	The sponsor of this trial, Centro Hospitalar Universitário Lisboa Central, E.P.E. (CHULC), included in the National Healthcare Service (*Serviço Nacional de Saúde*) The institutional Research Center “*Centro de Investigação”*, collaborated with the trial design, writing and the decision to submit the report for publication This is an investigator-initiated trial and the Principal Investigator is responsible for coordinating related all activities Principal investigator and corresponding author: Rui Antunes Pereira, email address rui.m.pereira@chlc.min-saude.pt, telephone contact + 351 934341322

## Introduction

### Background and rationale {6a}

Chronic liver disease patients with cirrhosis and ascites are at high risk for increased intra-abdominal pressure (IAP), and both the presence and the duration of intra-abdominal hypertension (IAH) are known independent risk factors for mortality in the critically ill patient [1, 2].

The prevalence of IAH and abdominal compartment syndrome (ACS) is very high among critically ill patients with liver cirrhosis when compared to other mixed populations of intensive care patients [3–5]. In the setting of IAH, paracentesis reduces abdominal wall tension and improves overall intra-abdominal hemodynamics by minimizing IAP and optimizing abdominal perfusion pressure (APP) of intra-abdominal organs [1].

Clinical practice guidelines for the critically ill patient with IAH/ACS, based on expert opinion, recognize liver dysfunction/cirrhosis with ascites as a risk factor and suggest the evacuation of obvious intra-peritoneal fluid with the use of percutaneous catheter drainage as part of a stepwise medical management algorithm to reduce and maintain IAP < 16 mmHg [1]. Nonetheless, IAH develops in more than three quarters of critically ill patients with cirrhosis during their first week of ICU stay [4, 5].

Therefore, a strategy for the prevention and treatment of IAH/ACS could improve patient outcomes in the critically ill cirrhotic patient by minimizing IAP and optimizing APP and potentially leading to improved multi-organic function. In fact, some studies in intensive care have shown that LVP is safe and associated with short-term improvement of renal, pulmonary, and hepatic dysfunction, although follow-up and clinical outcomes in the ICU were not assessed [4, 6–10]. Furthermore, therapeutic large-volume paracentesis (LVP) is the standard-of-care in the treatment of tense ascites and hepato-renal syndrome (HRS) in acutely decompensated liver cirrhosis patients [11–14]. An experimental study has demonstrated causality between small increases of IAP (5–10 mmHg), after merely 24 h, and the development of acute renal injury in HRS [15]. However, the recommendation for paracentesis in the treatment of IAH/ACS is based on low-quality evidence [1] and, to the best of our knowledge, there are no studies comparing different therapeutic paracentesis strategies in the critically ill cirrhotic patient with IAH [1].

### Objectives {7}

#### General objective

The general aim of this trial is to assess the impact of IAH on organ disfunction and clinical outcomes in the critically ill cirrhotic patient with ascites.

#### Specific objective

The objective of this trial is to compare the outcomes of two different methods of therapeutic paracentesis used in our clinical practice in the prevention and treatment of IAH, specifically, regarding the impact on renal function, as well as respiratory, neurological, cardiovascular, hematological, and hepatic functions, in addition to 28-day mortality and ICU length-of-stay (LOS).

## Trial hypothesis

A continuous passive paracentesis (CPP) strategy in the treatment of IAH improves organ dysfunction and clinical outcomes when compared to standard LVP in the critically ill cirrhotic with ascites.

## Methods

### Trial design {8}

#### Trial design

This is an investigator-initiated, single-site, open label, randomized clinical trial with parallel group allocation ratio of 1:1 to assess for efficacy and safety.

## Participants, interventions, and outcomes

### Study setting {9}

#### Trial settings

This trial is set in a general intensive care unit (ICU) specialized in liver disease at Hospital de Curry Cabral, Centro Hospitalar Universitário Lisboa Central (CHULC), Lisboa, Portugal, a tertiary university hospital, with an expected duration of 36 months.

### Eligibility criteria {10}

#### Population of interest

The critically ill patient presents with a life-threatening condition that requires pharmacological and/or mechanical support of vital organ functions﻿. The population of interest is the critical cirrhotic patient with ascites and acute decompensation admitted in the ICU.

#### Patient eligibility

All patients with liver cirrhosis admitted in the ICU stay are eligible for this trial.

#### Inclusion criteria

Inclusion criteria are defined by the following:

1. Adult patient (≥ 18 years old),
2. Diagnosis of liver cirrhosis and
3. Presence of ascites grade ≥ 2 [12].

#### Exclusion criteria

Exclusion criteria are defined by the following:

1. Extreme age (≥ over 75 years old),
2. Acute surgical condition or laparotomy in the preceding 4 weeks,
3. Previous liver transplant,
4. Hemorrhagic ascites (red blood cells count > 10,000/mL) [16],
5. Extreme clinical severity (APACHE II ≥ 34),
6. Any of the following conditions present 24 h after admission:
  a Hemorrhagic shock with active uncontrolled bleeding,
  b Refractory shock (mean arterial pressure < 65 mmHg) despite multiple vasopressor support,
7. Predictably short (< 72 h) ICU stay and
8. Therapeutic futility determined by the medical staff.

Notes:

- These criteria are to be applied independently of any previous treatments of ascites, including recent LVP (i.e., in the emergency department before ICU admission), and
- Examples of “predictably short (< 72 h) ICU stay, precluding patient enrolment, may be, i.e., the reversal of clinical instability after initial therapy and adequate resuscitation in the ICU, such as < 6 h of vasopressor support; variceal bleeding controlled in the ICU without developing organ failure; or prompt reversal of oliguria/AKI.

### Who will take informed consent? {26a}

The informed consent (Supplemental file 1) will be obtained by the attending physician from the proposed trial participant or authorized surrogate. In the particular case where the patient is not clinically able to decide (i.e., sedation or encephalopathy), and an authorized surrogate is not available, the Ethics Committee allowed for a presumed form of patient consent to participate in the clinical trial, based on public interest in the results of this study. The Principal Investigator (P.I.) and another ICU professional will endeavor any reasonable means to contact the authorized surrogate and provide written proof. Additionally, if at any time the patient, or it is authorized surrogate, is able to decide, then he/she may choose to withdraw consent to participate and be removed from the trial.

### Additional consent provisions for collection and use of participant data and biological specimens {26b}

There are no provisions for additional consent for ancillary studies using data not included in the original study protocol. Ancillary studies requiring additional data collection will be treated and conducted as independent studies.

## Interventions

### Explanation for the choice of comparators {6b}

This trial compares two methods of therapeutic paracentesis, namely, CPP versus standard-of-care LVP in the prevention and treatment of IAH. The CPP is expected to improve ascitic fluid drainage, therefore, providing better prevention and treatment of IAH, rather than intermittent LVP, which allows for the periodical accumulation of ascites with the potential deleterious effects of intermittent increases of IAP.

### Intervention description {11a}

The measurements of IAP will be performed every 6 to 8 h according to our center’s monitoring protocol (Supplementary file 2) and our clinical practice regarding the management of specific thresholds of IAP follows the current clinical guidelines to titrate therapy to maintain IAP less than 16 mmHg [1].

#### Control group—large-volume paracentesis

1. Ultrasound-guided (Ultrasound GE Vivid T8®) placement of an intra-abdominal catheter, 14 Gauge Optiva®, or similar, as indicated by the attending physician in accordance with international clinical guidelines [1, 12].
2. Collection of ascitic fluid for laboratorial and microbiological analyses with every new paracentesis.
3. Large-volume paracentesis will be performed as needed throught the ICU stay with a maximal duration of 8 h for each session.
4. The volume of drained ascitis through LVP is determined by the attending physician, according to the usual clinical practice, without specified maximum volume limit.

#### Intervention group—continuous passive paracentesis

1. Ultrasound-guided (Ultrasound GE Vivid T8®) placement of an intra-abdominal double lumen catheter, Kit Certofix® Duo720, or similar, for paracentesis.
2. Aseptic Seldinger technique, with an acute angle of percutaneous needle insertion.
3. Adhesive, suture-free, abdominal wall catheter fixation.
4. Continuous passive ascitic fluid drainage, preferentially through the proximal lumen (lateral opening) to minimize obstruction.
5. Collection of ascitic fluid every 48 h for laboratorial and microbiological analyses.
6. The trial intervention catheter will be in place for a maximum duration of 7 days.
7. The catheter should be removed after 7 days of intervention or prior to ICU discharge, whatever occurs first.
8. After the 7th day of intervention, paracentesis should be performed using the standard LVP approach as needed.

The 7-day duration of the intervention was determined based on previous studies revealing that the cumulative prevalence of IAH reaches a relative steady-state 5 days after ICU admission, with a median ICU stay of between 5 and 8 days in the studied populations [17, 18]. Furthermore, the impact of IAH on renal disfunction, assessed by the rise in serum creatinine, may lag up to approximately 3 days [19]. Therefore, we considered reasonable a period of 7 day for the trial intervention, while minimizing the risk of paracentesis catheter-associated infection, albeit considered safe [20–22].

### Criteria for discontinuing or modifying allocated interventions {11b}

The attending physician’s judgment is definitive regarding all clinical decisions, including protocol modification, interruption, or discontinuation, and these should be clearly stated in the patient’s clinical record.

In case of signs of catheter-related complication, particularly in the intervention group, appropriate therapeutic measures should be taken and the paracentesis catheter should be replaced when feasible at a different site for continued protocol intervention.

### Strategies to improve adherence to interventions {11c}

Strategies to improve adherence to the trial protocol include regular sessions and briefings with the nursing and medical staff throughout the duration of the trial addressing patient recruitment and enhanced protocol compliance, focusing on IAP and APP monitoring , 8 h urine sample collection procedures, and trial-related interventions.

### Relevant concomitant care permitted or prohibited during the trial {11d}

There are no restrictions to patient care during this trial.

#### Safety procedures and assessment

Clinical management will comply with current guidelines, including strict prevention, detection, and treatment of paracentesis-related complications, including paracentesis associated circulatory dysfunction, hypovolemia, intra-abdominal organ perforation and bleeding, catheter-related abdominal wall infection, and secondary peritonitis and abdominal wall fistula or bleeding [12]. Paracentesis-related complications and harm will be actively screened and reported by clinicians.

Paracentesis associated circulatory dysfunction is prevented with standard-of-care intravenous 20% albumin infusion (8 g for each 1 l of drained ascitic fluid) administered to all patients and, additionally, crystalloid infusion may be indicated by the attending physician to strictly avoid hypovolemia [12].

Intra-abdominal organ perforation and bleeding are prevented with (a) ultrasound guided paracentesis and (b) coagulopathy treatment, mandatory in case of severe thrombocytopenia (platelet count < 50,000/mL) or hypofibrinogenemia (< 0.7 g/L), as clinically indicated by the attending physician or the Blood Transfusion department [23–27].

Catheter-related abdominal wall infection and secondary peritonitis are to be prevented with aseptic paracentesis technique and standard catheter point of insertion surveillance for signs of inflammation or infection, similar to central venous catheter procedures [28].

We do not use prophylactic antibiotics along with the indwelling abdominal paracentesis catheter (IAPC) in the CPP intervention group nor in the control group.

The definition of secondary peritonitis includes the following: (1) polymorphonuclear cell count elevation > 50% in 48 h after the initial paracentesis or (2) microbiologic “de novo” isolation of bacteria after 48 h after paracentesis.

In case of isolated bacterial culture of suspected skin contaminants, without signs of infection, a new direct ascitic tap at a different site should be performed to confirm or exclude a diagnosis of secondary peritonitis.

Whenever a clinical diagnosis of secondary peritonitis is considered, then antibiotic treatment should be immediately started according to standard clinical practice.

In case of paracentesis catheter-associated infection in the intervention group, similar to catheter-related bloodstream infection diagnosis and treatment, the catheter must be removed and, when clinically feasible, replaced via a new direct ascitic tap at a different site [28]. This will allow for ascitic fluid analysis after 48 h to monitor for treatment response, according to clinical guidelines, and to complete the 7-day period of the trial intervention [12].

If abdominal wall fistula or bleeding occurs, the insertion point should be sutured for closure and hemostasis.

### Provisions for post-trial care {30}

The proposed interventions are to be, exclusively, implemented in the intensive care setting, although both post-trial care and patient follow-up will continue up to hospital discharge.

Patients enrolled in this study are covered from negligence or harm by the clinical trial insurance contracted by the study sponsor (CHULC, reference no. 706/22).

### Outcomes {12}

The primary outcome is serum creatinine concentration. The secondary outcomes are IAP, measured creatinine clearance, daily urine output, the multiorgan disfunction score Chronic Liver Failure Sequential Organ Failure Assessment (CLIF-SOFA) [13] and the incidence of stage 3 acute kidney injury (AKI) (defined by Kidney Disease: Improving Global Outcomes—KDIGO AKI [29] score), as well as 28-day renal replacement therapy (RRT)-free days, 28-day survival rate, and ICU LOS. The outcomes variables will be assessed at day seven (D7) after randomization (D0), unless otherwise stated. Additionally, IAP, serum creatinine concentration, measured creatinine clearance, daily urine output, and CLIF-SOFA will be analyzed using their maximum or minimum and daily mean values between D1 and D7.

Whenever ICU discharge or liver transplant occurs before D7, the last available data prior to these events will be used for outcome assessment. Additionally, stage 3 AKI will be considered when RRT or death occurs and, in these circumstances, a serum creatinine concentration value of 4 mg/dL and creatinine clearance value of 10 mL/min will be used for outcome assessment as mentioned in the literature [13, 29, 30].

### Participant timeline {13}

The participants’ timeline begins with the screening for eligibility at ICU admission, obtainment of informed consent, enrolment, and randomized trial group allocation during the initial 24 h of stay. The calendar day in which group allocation takes place is considered trial “day zero” (D0).

The placement of the abdominal double-lumen catheter is intended to be performed as soon as possible after the allocation in the intervention group in order to optimize IAP and prevent and treat IAH. This procedure does not require special training beyond standard ultrasound-guided Seldinger technique.

Unforeseen catheter placement delay of more than 24 h after group allocation without clinical justification will lead to patient exclusion from the trial.

Protocol interventions and measurements will be maintained up to D7, after which post-trial care and follow-up will ensue until hospital discharge. This trial is expected to complete recruitment and follow-up of 70 patients in 36 months, until July 2025.

### Sample size {14}

The sample size was calculated using the statistical noncentral *t *function, with a one-sided alpha level of 0.05 [31]. The calculations were based on the expected renal function and IAP variation between groups, since we assume the former to be dependent of the latter.

To support the trial rationale of an expected clinically significant IAP decrease of 3 mmHg in the intervention group, a total of 44 patients are considered necessary to detect a statistically significant difference, with 1:1 allocation ratio, 80% statistical power, and 95% confidence level. This estimate is based in a post-hoc analysis of 61 patients with cirrhosis from a multicentric randomized controlled trial in shock patients where a mean difference in IAP of 3 mmHg was found between survivors and non-survivors (13.7 ± 3.9 and 16.7 ± 3.9 mmHg, respectively) [4].

Regarding the primary outcome, to detect a clinically and statistically significant decrease in serum creatinine of 0,4 mg/dL in the intervention group, a total of 60 patients is estimated, with a 1:1 allocation ratio, 95% statistical power, and 95% confidence. This estimate is based on a multicentric observational study of acutely decompensated patients with cirrhosis. In this study, cirrhotic acute-on-chronic liver failure patients with at least one organ failure had a mean creatinine difference of 0,4 mg/dL between survivors and non-survivor groups (0.9 ± 0.45 and 1.3 ± 0.48 mg/dL) [32].

We arbitrarily estimate that protocol non-adherence/attrition or violation after randomization may affect approximately 15% of cases and, therefore, to ensure that the number of patients that complete the protocol is reached for the primary outcome, the total sample size is set at 70 patients with a 1:1 group allocation ratio.

### Recruitment {15}

To optimize patient enrollment, nursing and medical staff will screen all admissions for patients with cirrhosis into the ICU and signal them to the P.I. for timely recruitment.

## Assignment of interventions: allocation

### Sequence generation {16a}

The patient allocation method consists of stratified randomization with blocks within each subgroup. This method is particularly useful with randomizing small samples due to the ability to create balanced groups regarding predetermined variables or characteristics at the beginning of the trial, reducing differences in baseline trial groups characteristics’ that could harm later result analysis and conclusions.

In this small sample size trial, patients will be stratified according to maximum serum creatinine and maximum IAP, expected to be the two most important variables to balance between trial groups at baseline. Stratified randomization will homogenize trial groups ad initio to reduce the probability of unbalanced baseline group characteristics and biased results.

The stratification process will use two strata, namely, maximum IAP and maximum serum creatinine, thus creating four subgroups (I–IV): (I) IAP < 16 mmHg + serum creatinine < 1.5 mg/dL, (II) IAP < 16 mmHg + serum creatinine ≥ 1.5 mg/dL, (III) IAP ≥ 16 mmHg + serum creatinine < 1.5 mg/dL, and (IV) IAP ≥ 16 mmHg + serum creatinine ≥ 1.5 mg/dL [4, 13]. Each subgroup (I–IV) contains sequential blocks of randomized trial group allocation (A) control and (B) intervention, with a 1:1 ratio (i.e., *AABB*, *ABAB*, *BABA*, *BBAA*), as exemplified in Table 1.

**Table 1:** Stratification subgroups (I-IV) created using maximum intra-abdominal pressure (IAP) and serum creatinine as strata. Example of stratified randomization with blocks within each subgroup (A, B)

Stratification subgroups (I–IV)	Creatinine < 1.5 mg/dL	Creatinine ≥ 1.5 mg/dL
IAP < 16 mmHg	I) ABAB, ABBA,…	III) BABA, BBAA,…
IAP ≥ 16 mmHg	II) BABA, AABB,…	IV) BBAA, BAAB,…

### Concealment mechanism {16b}

The adopted concealment mechanism of the allocation sequence will use sequentially numbered (#), opaque, sealed envelopes, and the random sequence for trial group allocation in blocks will be generated by the P.I. using an online tool [33].

To prevent biased selection of patients, the PI will remotely screen for new patient admissions and alert the ICU staff for possible candidates. The patient’s attending physicians are responsible for applying protocol, deciding patient eligibility, and checking for inclusion/exclusion criteria and obtaining the informed consent.

Once patient enrollment has been established, the attending physicians, who are blinded for the allocation blocks, will determine the corresponding stratification subgroup by using IAP and creatinine values and open the respective sealed envelope containing the group allocation. The PI will provide any required assistance, and, finally, be informed of the patient allocation.

### Implementation {16c}

The allocation mechanism will be implemented by the patient’s attending physician, after determining the stratification group (I–IV) by opening the corresponding envelope with the lowest available number (#).

## Assignment of interventions: blinding

### Who will be blinded {17a}

Given the open nature of the trial, the assigned interventions will be unblinded for the clinical staff and patient. Outcome assessment and data analysts will also be unblinded for patient’s group allocation given the objective character of the outcomes and the open trial intervention.

### Procedure for unblinding if needed {17b}

Not applicable as no blinding was used in this trial.

## Data collection and management

### Plans for assessment and collection of outcomes {18a}

The assessment and collection of trial data will start at patient enrollment (D0) including vital signs, specific therapies (i.e., albumin, transfusions), vital organ support, blood, urine, and ascitic fluid tests, repeatedly up to D7.

The trial protocol includes the daily collection of an 8-h (480 min) urine volume, at blocked nocturnal interval from 23 to 07 h, for measurement of creatinine clearance, based on the following formula: creatinine clearance (mL/min) = (urinary creatinine [mg/mL] × urine volume [mL])/(serum creatinine [mg/mL] × urine collection time [480 min]) [34].

Daily blood analysis includes complete hemogram, coagulation, and biochemistry and arterial blood gas. Ascitic fluid will be collected with every new paracentesis and repeated every 48 h in the intervention group for screening of infectious complications.

Collected trial variables are mainly included in clinical severity scores APACHE II [35], SAPS II [36], CLIF-C ACLF [37], CLIF-SOFA, SOFA [38], RIFLE [30], KDIGO [29], ICA-AKI [13], MELD [39], and MELD-Na [40] and incorporate liver disease etiology, precipitant event for critical illness, urine output, serum creatinine, estimated and measured creatinine clearance, and number of days on RRT, Glasgow coma score and West-Haven scale, number of days under vasopressor support and dosage, PaO_2_/FiO_2_ ratio, positive end-expiratory pressure and number of days under mechanical ventilation, total bilirubin, coagulation international normalized ratio, platelet count, arterial blood lactate and prescribed albumin dosage. Intermediate effect variables IAP and APP will be measured throughout ICU stay to test the trial rationale.

### Plans to promote participant retention and complete follow-up {18b}

The P.I. will promote participant retention and complete follow-up by daily checking with the clinical staff for new eligible patients, assuring protocol adherence and complete follow-up during the ICU and hospital stays of enrolled patients.

### Data management {19}

Patient identification will be coded and pseudo-anonymized in a list kept confidential by the P.I. up to 1 year after publication of trial results, after which it will be destroyed.

Data will be collected into a database (IBM Corp. Released 2020. IBM SPSS Statistics for Windows, Version 27.0. Armonk, NY: IBM Corp), located in a secure institutional space, accessible only through specific ID and passwords, restricted to the P.I. and associated investigators.

The P.I. will be responsible for collected data quality and the database safeguard for a period of 10 years after patient enrollment is complete.

### Confidentiality {27}

The coded list will be used for patient confidentiality and pseudo-anonymization, and to allow for quality control, while managing and analyzing the database. It will be kept in a secure institutional space, accessible only through specific ID and passwords.

### Plans for collection, laboratory evaluation, and storage of biological specimens for genetic or molecular analysis in this trial/future use {33}

This trial does not include any collection of biological specimens for genetic or molecular analysis.

## Statistical methods

### Statistical methods for primary and secondary outcomes {20a}

For the descriptive analysis, quantitative variables will be reported as means and standard deviations or as medians and interquartile ranges (*P*_25_-*P*_75_), as appropriate, and categorical variables reported as frequencies and percentages.

Quantitative variables will be analyzed using the assessment day value (D7), the daily mean value, and their maximum or minimum values. Mann–Whitney *U* test will be used to compare study groups, assuming a non-normal distribution, or two independent sample *t*-test, in case of normal distribution. Dichotomous categorical variables will be analyzed using chi-square or Fisher’s exact tests. Using daily values, mixed effects regression models will be used to consider the autocorrelation structure between the longitudinal measurements. Regarding stage 3 AKI as secondary outcome, odds ratios and corresponding 95% confidence intervals will be estimated using logistic regression models. For the study of RRT-free days, competing risks survival models will be applied considering death as the competing risk. Regarding time until death, joint survival regression models will be used to take into consideration the association of longitudinal markers with time until death. For LOS, linear regression models will be applied. For all regression studies, univariable and multivariable analyses will be performed to account for potential confounders. The absolute risk of paracentesis-related complications will be reported for both trial groups. A level of significance *α*= 0.05 will be considered. Data will be analyzed using the R Statistical Software [41].

### Interim analyses {21b}

An interim analysis will be performed when the trial completes the follow-up of 30 patients to assess for safety and outcomes. The trial will be terminated in case of significant harm or if significant statistical difference on the primary outcome is achieved.

### Methods for additional analyses (e.g., subgroup analyses) {20b}

Subgroup analyses are planned to compare the outcomes of groups of patients with or without serum maximum creatinine ≥ 1.5 mg/dL, maximum or mean IAP value ≥ 16 mmHg before randomization, and baseline or mean APP value < 60 mmHg up to D7.

### Methods in analysis to handle protocol non-adherence and any statistical methods to handle missing data {20c}

Data will be analyzed according to intention-to-treat (ITT). Protocol adherence, with intervention initiation and completeness, will be reported. Missing data will not be imputated and will be described.

### Plans to give access to the full protocol, participant level-data and statistical code {31c}

Access to trial data will be made available upon reasonable request to the PI.

## Oversight and monitoring

### Composition of the coordinating center and trial steering committee {5d}

The trial coordination will be in charge of the P.I. and a steering committee will include two additional collaborators.

### Composition of the data monitoring committee, its role and reporting structure {21a}

A data monitoring committee (DMC) composed of three independent external collaborators, including a statistician, will report to the PI and the Research Center on aspects of trial conduct, such as recruitment, identify the need to make adjustments, and analyze for significant outcomes or harm.

### Adverse event reporting and harms {22}

Should any adverse events, unintended effects, or harm be detected, it is the attending physician’s responsibility to report to the PI and the Investigation Centre of CHULC.

### Frequency and plans for auditing trial conduct {23}

The DMC is expected to perform  trial conduct audits once 20 and 40 randomized patients have completed 28-day follow-up and at trial conclusion.

### Plans for communicating important protocol amendments to relevant parties (e.g., trial participants, ethical committees) {25}

Any protocol modifications or amendments must be communicated to the Research Center and Ethics Committee of CHULC.

### Dissemination plans {31a}

The dissemination of the trial results will take place in the form of public presentations and publications in the appropriate scientific media.

## Discussion

This is the first trial to compare the impact of different therapeutic paracentesis strategies on organ dysfunction and outcomes in the prevention and treatment of IAH in critically ill patients with cirrhosis and ascites. Ultimately, we expect to discuss these strategies and improve clinical practice.

Our center has experience in treating critically ill patients with cirrhosis and ascites and IAH and we use both LVP and CPP strategies empirically. The CPP has the potential to minimize IAP, optimize APP, and reduce the number of required abdominal punctures and the risk of perforation and bleeding. However, it may entail higher risk of infection and abdominal wall fistula, when compared to intermittent LVP. The need to better understand the impact of these interventions set the grounds for this trial.

The use of a double lumen catheter for continuous ascitic fluid drainage presents important advantages over a single lumen catheter because: (1) it is more resistant to obstruction by “kinking” due to its internal section pillar and (2) the proximal (lateral) opening is less prone to obstruction by viscera or clotting than the distal (pointing) one. Furthermore, the use of the Seldinger technique to insert the abdominal paracentesis catheter, using an acute angle of percutaneous insertion (ideally, less than 45°), avoiding a perpendicular approach, (1) reduces catheter kinking and (2) may prevent insertion site fistula, given the oblique multiplane closure trajectory. These details are even more important given the duration of the CPP strategy.

The use of IAPC in patients with cirrhosis and ascites has been demonstrated to be safe and effective outside of the ICU setting [20–22]. We do not use prophylactic antibiotics associated with paracentesis in this trial nor in our usual clinical practice. Similarly, in the prevention of catheter-related bloodstream infections, the use of prophylactic antibiotics is not recommended in the clinical management of non-tunneled central venous catheters [28]. Additionally, the use of prophylactic antibiotics in the intervention arm would create a important therapeutic differences between study groups and hinder the comparison of results.

Possible limitations in our trial protocol include the following: (1) underpowered sample size to identify significant differences for some secondary outcomes and paracentesis-related complications or harm; (2) reduced generalizability of results due to the single-center, specialized liver disease ICU setting; (3) selection bias due to the fixed block size randomization; for this reason, the group allocation will be revealed by the attending physician, rather than the P.I.; (4) clinical reasons may induce protocol non-adherence or violation, although this may reflect real-world conditions and improve generalizability of results.

Finally, we chose serum creatinine as the primary outcome variable to compare trial groups since it remains the most practical biomarker of renal function in patients with AKI [13]. Even though serum creatinine has some limitations, particularly regarding interpretation in the individual cirrhotic patient, the randomization process should homogenize patient characteristics at baseline and allow for the comparison of results between groups.

This research trial conforms with the ethical norms and standards in the Declaration of Helsinki, including local Ethics Committee (reference no. 632/2018, 11/02/2022) and registration (ClinicalTrials.gov NCT04322201). This trial has received internal funding granted from the Research Center (reference FFI 06/2019), exclusively for specific laboratory analysis described in the trial protocol.

## Trial status

The present trial protocol corresponds to version 10.4, January 2022, and has been implemented in August 2022. The recruitment process began with the enrolment of the first patient in September 2022 and is expected to be complete in 36 months, by July 2025.

Trial registration: ClinicalTrials.gov NCT04322201. Registered 20/12/2019, https://trialsearch.who.int/Trial2.aspx?TrialID=NCT04322201.

### Supplementary Information

**Additional file 1. **Informed consent.

**Additional file 2. **Out center’s monitoring protocol.

## Data Availability

The PI will oversee the study data sharing processes during and after the trial is complete. To ensure confidentiality, shared data will be blinded of any identifying participant information. The study dataset and materials will be password protected and safeguarded by the PI. The final dataset will be made available upon reasonable request.
