# Supplementary material for: Continuous passive paracentesis versus large-volume paracentesis in the prevention and treatment of intra-abdominal hypertension in the critically ill cirrhotic patient with ascites (COPPTRIAHL): study protocol for a randomized controlled trial
Source: Trials. 2023 Aug 15;24:534. doi: 10.1186/s13063-023-07541-4 (PMC10426145; doi:10.1186/s13063-023-07541-4)
Supplement: Supplementary file 1 — Additional file 1. Informed consent. [file 13063_2023_7541_MOESM1_ESM.pdf]

CENTRO HOSPITALAR DE LISBOA CENTRAL, EPE

INFORMAÇÃO PARA OBTENÇÃO DE CONSENTIMENTO ESCLARECIDO PARA PARTICIPAÇÃO EM ESTUDOS DE INVESTIGAÇÃO

A relação investigador-participante é baseada na confiança mútua e está protegida por lei. O investigador obriga-se a informar o participante ou o seu representante legal sobre a natureza da sua participação no estudo, potenciais vantagens e inconvenientes, podendo o mesmo aceitar ou não participar no estudo. Por favor, leia com atenção todo o conteúdo deste documento. Não hesite em solicitar mais informações se não estiver completamente esclarecido/a.

Título do estudo: OPTIMIZAÇÃO DA PRESSÃO DE PERFUSÃO ABDOMINAL PELO CONTROLO DA PRESSÃO INTRA-ABDOMINAL NO DOENTE COM CIRROSE HEPÁTICA EM CUIDADOS INTENSIVOS.

Áreas/Unidades: UCIP7 , Hospital Curry Cabral , CHULC

Descrição do estudo:

A cirrose do fígado produz ascite (líquido dentro da barriga) e aumento excessivo da tensão na barriga (pressão intra-abdominal) que pode necessitar de tratamento. O tratamento inclui a **paracentese** (picar a barriga para que retirar a ascite).

Atualmente, o tratamento habitual é a **paracentese de grande volume,** repetida conforme a indicação do médico.

Este estudo clínico com intervenção compara duas técnicas de paracentese para determinar qual a mais eficaz e segura para tratar os doentes cirróticos em Cuidados Intensivos

Assim, iremos comparar a **paracentese contínua passiva** mantida durante todo o internamento em Cuidados Intensivos com o tratamento habitual. O potencial benefício desta inclui reduzir o número de punções da ascite e melhorar o

funcionamento dos órgãos intra-abdominais. As duas técnicas têm os mesmos riscos, próprios da paracentese: infeção e hemorragia, que serão vigiadas e prevenidas da forma habitual.

Os doentes que participem no estudo são sorteados para ser tratados com uma ou a outra técnica, **sem prejuízo do seu tratamento**, e terão um **seguro de proteção** próprio.

Este estudo clínico com intervenção tem o objetivo de melhorar o tratamento dos doentes cirróticos mais graves, faz parte de um programa de doutoramento pela Nova Medical School da Universidade Nova de Lisboa, é apoiado pelo Centro Hospitalar de Lisboa Central e não prevê qualquer tipo de financiamento externo.

A participação no estudo é totalmente **voluntária**. A participação, ou a recusa em participar, não afeta a qualidade dos tratamentos e dos cuidados prestados ao doente.

Os dados clínicos são recolhidos do processo clínico e incluem idade, sexo, história de doenças, sinais vitais e análises, são **anónimos** e **confidenciais**, respeitando o Regulamento Geral sobre a Proteção de Dados de 25 de maio de 2018.

Em caso de o doente não saiba/consiga assinar poderá dar o seu consentimento oral na presença de uma testemunha que assinará.

Parte declarativa do profissional de saúde

Nome: ____

Número da ordem: ______________   Data: ____/____/_____

Confirmo que expliquei à pessoa abaixo indicada, de forma adequada e inteligível, os procedimentos necessários ao ato referido neste documento. Respondi a todas as questões que me foram colocadas e assegurei-me de que houve um período de reflexão suficiente para a tomada da decisão. Também garanti que, em caso de recusa, serão assegurados os melhores cuidados possíveis nesse contexto, no respeito pelos seus direitos. Em caso de dúvidas contactar a UCIP7 (217924271).

____

Parte declarativa do utente.

Declaro ter compreendido os objetivos de quanto me foi proposto e explicado pelo profissional de saúde que assina este documento, ter-me sido dada oportunidade de fazer todas as perguntas sobre o assunto e para todas elas ter obtido resposta esclarecedora, ter-me sido garantido que não haverá prejuízo para os meus direitos assistenciais se eu recusar esta solicitação, e ter-me sido dado tempo suficiente para refletir sobre esta proposta.

☐ Autorizo participar neste estudo clínico com intervenção.

☐ Não autorizo participar neste estudo clínico com intervenção.

Documento de identificação:________________

Assinatura:______________________________ Data da assinatura: ____/____/_____
