# Supplementary material for: Continuous passive paracentesis versus large-volume paracentesis in the prevention and treatment of intra-abdominal hypertension in the critically ill cirrhotic patient with ascites (COPPTRIAHL): study protocol for a randomized controlled trial
Source: Trials. 2023 Aug 15;24:534. doi: 10.1186/s13063-023-07541-4 (PMC10426145; doi:10.1186/s13063-023-07541-4)
Supplement: Supplementary file 2 — Additional file 2. Out center’s monitoring protocol. [file 13063_2023_7541_MOESM2_ESM.pdf]

# Protocolo de monitorização de Pressão Intra-Abdominal

**Elaboração**

Dr. Rui Pereira, Enf. Vitor Inácio, Enf. Duarte Mateus e Enf. Vitor Almeida (UCIP7, HCC, CHULC)

**Objetivo**

Protocolo de procedimento para colocação de sistema de monitorização da pressão intra-abdominal e manutenção do sistema.

**Siglas e Abreviaturas**

PIA - Pressão Intra-Abdominal

HIA - Hipertensão Intra-Abdominal

SCA - Síndrome de Compartimento Abdominal

PPA - Pressão de Perfusão Abdominal

mmHg - milímetros de mercúrio

UCIP - Unidade de Cuidados Intensivos

TA - tensão arterial

# Definições

## Pressão Intra-Abdominal

O valor normal da PIA situa-se entre 5-7 mmHg e considera-se patológico quando é igual ou superior 12 mmHg de forma repetida ou mantida.

A medição da PIA é indireta, via vesical, com instilação de 25 cc de soro através da algália, como doente em decúbito supino, relaxado, com o nível zero de pressão do sistema da linha média axilar do doente, correspondente ao eixo flebostático, tal como para a medição da pressão arterial.

## Hipertensão Intra-Abdominal

A HIA define-se pela manutenção de valores de PIA igual ou superior a 12 mmHg.

A classificação da HIA apresenta quatro graus (I-IV) de acorodo com o valor de PIA: grau I: 12-15 mmHg; grau II: 16-20 mmHg; grau III: 21-25 mmHg; grau IV: > 25 mmHg.

A HIA pode ser **primária** (processo patológico de origem abdomino-pélvica), **secundária** (processo patológico de origem extra cavidade abdomino-pélvica) ou **recorrente** (quando reaparece após terapêutica do processo de HIA).

## Síndrome de Compartimento Abdominal

A SCA define-se por um valor PIA igual ou superior a 20 mmHg associado a disfunção orgânica de novo.

**Pressão de Perfusão Abdominal**

A PPA reflete o gradiente de pressão entre o sistema arterial e venoso que mantém o fluxo sanguíneo através dos órgãos intra-abdominais.

A fórmula da PPA é a seguinte: PPA = TA média - PIA

O valor de PPA considerado normal é igual ou superior 60mmHg. Valores inferiores a 55 mmHg estão associados com aumento de mortalidade.

**<u>Descrição</u>**

1. A presença de PIA elevada em 3 mensurações seguidas por avaliação indirecta com intervalos de 4/6h reforça a sua fiabilidade.
2. As indicações para a avaliação da PIA incluem:
    a. - Trauma abdominal (ex. fechado ou aberto, hemorragia retroperitoneal, fraturas pélvicas, queimaduras extensas);
    b. - Cirurgia abdominal major (ex. neoplasias, pancreatite, oclusão intestinal, rotura de aneurismas da aorta abdominal)
    c. - Patologia médica (ex. choque séptico, ascite, obesidade mórbida)
3. São contra-indicações para a medição da PIA as situações como rotura vesical e bexiga neurogénica.
4. Todas as intervenções para colocação, manutenção e avaliação da PIA devem respeitar a técnica asséptica.
5. A preparação do material para a avaliação da PIA deve incluir:

   a. - Módulo de avaliação de pressão e cabo de monitorização.
   b. - Sistema com Soro Fisiológico a 0.9% (500cc).
   c. – Sistema de vias de pressão.
   d. - Conector 3/8x1/4LL (Maquet (R)
   e. - Seringa de 50 mL.
   f. - Material de desinfeção (compressas, luvas. campo esterilizado, álcool 70%).
6. A montagem do sistema de monitorização da PIA:
   a. -- Adaptar o conector 3/8x1/4 entre a algália e o sistema de medição horária de diurese.
   b. -- Adaptar sistema de vias de pressão entre o Conector 3/8x1/4LL (Maquet (R) e módulo de avaliação de pressão.
   c. -- Conectar Soro Fisiológico a 0.9% (500cc) ao módulo de avaliação de pressão.
   d. – Configurar o monitor para medição de PIA utilizando o modo de “PVC” (pressão venosa central).
7. -- Proceder à avaliação da PIA de acordo com a indicação clínica.
8. O procedimento de avaliação da PIA consiste em:
   a. - A unidade de medição é "mmHg".
   b. - Avaliação da PIA de forma intermitente.
   c. - Avaliação da TA média simultaneamente com a PIA
   d. - Selecionar no monitor do doente o módulo de "Pressão venosa central", que serve igualmente para a monitorização da PIA.

e. - Posicionar o doente, relaxado, em decúbito dorsal com a cama a zero graus (0º) de inclinação.
f. - Colocar o transdutor da PIA e da TA ambos ao nível da linha média axilar, no eixo flebostático, de preferência dentro do leito do doente para minimizar desvios.
g. - Clampar o sistema de dispositivo urinário para evitar fuga do soro instilado para o dispositivo coletor e erro de medição da PIA.
h. - Realizar a calibração do nível "zero" de pressão do sistema de monitorização da PIA e da TA.
i. - Desinfetar a torneira do sistema de monitorização de pressão com álcool 70%.
j. - Com a seringa de 50 mL, aspirar 25 mL de solução de cloreto de sódio a 0,9%, do sistema de monitorização de pressão e injetar na cavidade vesical.
k. - Ligar o sistema de pressões à cavidade vesical e aguardar 30-60 segundos pelo valor numérico associado à presença de uma curva de pressão, caracteristicamente pouco oscilante.
l. - Considerar o valor médio da PIA (a medição da PIA deve considerar o momento final da expiração do doente, o valor habitualmente mais baixo).
m. - Desclampar o sistema após a avaliação

9. Registar o valor da PIA no SClínico.
10. Registar o valor da TA média no SClínico.

11. O volume de cloreto de sódio 0,9% instilado na bexiga para o procedimento deve ser subtraído ao débido urinário dessa hora.
12. Se valores de PIA iguais ou superiores a 12mmHg, deve repetir-se o procedimento de 4/4h.
13. Vigiar complicações da monitorização da PIA (infeção de trato urinário), equivalentes às da algaliação do doente.
14. Na manutenção do sistema para avaliação da PIA deve-se:
15. Substituir todo o sistema a cada 72 horas.
16. Identificar o sistema de PIA e o módulo.

**<u>Referências bibliográficas</u>**

Kirkpatrick et al. Intra-abdominal hypertension and the abdominal compartment syndrome: updated consensus definitions and clinical practice guidelines from the World Society of the Abdominal Compartment Syndrome. Intensive Care Med. 2013 Jul;39(7):1190-206.
